# Role of the complement anaphylatoxin C5a-receptor pathway in atopic dermatitis in mice

**DOI:** 10.3892/mmr.2015.3301

**Published:** 2015-02-04

**Authors:** LIN DANG, LEI HE, YAN WANG, JIKUI XIONG, BINGXUE BAI, YUZHEN LI

**Affiliations:** 1 Department of Dermatology, The Second Affiliated Hospital of Harbin Medical University, Harbin, Heilongjiang 150086, P.R. China; 2 Department of Pathology, Heilongjiang Provincial Hospital, Harbin, Heilongjiang 150001, P.R. China

**Keywords:** atopic dermatitis, C5a receptor antagonist, mast cell, interleukin-4, interferon-γ, histamine, immunoglobulin E

## Abstract

Atopic dermatitis (AD) is a chronic inflammatory skin disease with a genetic background. The C5a-receptor (C5aR) pathway has been reported to be involved in AD; however, the precise pathogenesis remains to be elucidated. In the present study, the contribution of the C5aR pathway to AD in mice was investigated. A BALB/c mouse model of AD was induced by application of 2,4-dinitrochlorobenzene (DNCB) onto hairless dorsal skin. Following DNCB application for 2 weeks, C5aR expression in skin tissue was assessed by reverse transcription quantitative polymerase chain reaction. C5aR expression in skin tissue was significantly increased in mice with AD. In an additional experiment, C5aR antagonist (C5aRA) intracutaneously injected in combination with DNCB treatment. The skin-fold thickness, number of total infiltrating leukocytes and mast cells infiltrating in skin tissue were measured. Interleukin-4 (IL-4) and interferon-γ (IFN-γ) levels in skin tissue and IL-4, IFN-γ, histamine and immunoglobulin E (IgE) levels in serum were measured using ELISA. The skin-fold thickness, numbers of total infiltrating leukocytes and mast cells in skin tissue, as well as levels of IL-4, IFN-γ, histamine and IgE were significantly increased in mice with AD. However, simultaneous treatment with C5aRA significantly attenuated increases in skin fold thickness and the numbers of total infiltrating leukocytes and mast cells in skin tissue. Treatment with C5aRA also decreased IL-4 and IFN-γ levels in skin tissue, as well as the levels of IL-4, IFN-γ, histamine and IgE in the serum. In conclusion, C5aRA inhibited AD in mice, possibly through suppression of the C5aR-mediated cascade action of mast cells.

## Introduction

Atopic dermatitis (AD) is an inflammatory skin disease, which is associated with allergic diseases and has a genetic background (1). Atopic dermatitis occurs mainly in response to an infiltration of mast cells (2) and dysregulation in T helper (Th) cell reactivities (3). The production of interleukin-4 (IL-4) from Th2 cells and mast cells and interferon-γ (IFN-γ), which is produced from Th1 or mixed Th1/Th2 cells, has been observed to trigger isotype-switching to immunoglobulins in AD (4). Excessive immunoglobulin E (IgE) production and histamine release from activated mast cells are indicative of AD in humans and have been observed in mice with AD-like skin lesions (5,6). Skin mast cells contribute to AD inflammation. The increased levels of IL-4 and IFN-γ, IgE production and histamine release during AD are all closely associated with the infiltration of mast cells into the site of inflammation (2). Mast cells are involved in AD through releasing Th2 polarizing cytokines, IL-4 and generating pruritus symptoms through release of histamine and tryptase (7). In addition, mast cells may increase histamine release and enhance IgE-dependent skin inflammation in mice (8).

Previous studies on complement anaphylatoxin receptors have been undertaken in multiple fields, including cardiovascular disease and inflammation (9–11). C5a is a risk factor for skin inflammation and specifically binds to its receptor C5aR on immune cells, including mast cells and leads to proinflammatory activation (12). It has been demonstrated that C5a can induce chemoattraction and induce secretion in mast cells during inflammation through a C5aR-mediated pathway (13); therefore, the aim of the present study was to determine whether a C5aR antagonist (C5aRA) inhibits AD in mice through suppressing the C5aR-mediated cascade action of mast cells.

In the present study, the role of C5aR-mediated activity during AD was assessed for the first time, to the best of our knowledge. Dorsal skin of BALB/c mice was treated with 2,4-dinitrochlorobenzene (DNCB) and C5aR expression in skin tissue was assessed. The effect of DNCB or DCNB combined with C5aRA on skin-fold thickness the number of total infiltrating leukocytes (mast cells, neutrophils, lymphocytes, monocytes and a few eosinophils and basophils) as well as the levels of IL-4, IFN-γ, histamine and IgE was also in determined. The results indicated that C5aRA inhibited AD in mice, possibly through suppressing the C5aR-mediated cascade action of mast cells, which may represent a novel therapeutic strategy for AD.

## Materials and methods

### Animals

BALB/c mice (specific pathogen-free males, 7 weeks old) were purchased from Shanghai Laboratory Animal Centre (Shanghai, China). The animal care and protocol of the present study was in accordance with the Animal Experiment Guidelines of Harbin Medical University (Harbin, China). The mice were kept under a 12-h light/dark cycle at 22°C and fed an unlimited quantity of water and feed throughout the duration of the experiment. The mice were divided into three groups: Control group, AD-like symptom group and C5aRA-treated group.

### Induction of an atopic dermatitis-like skin disorder with corresponding immunology

DNCB was applied onto the hairless dorsal skin of the mice. Following complete removal of dorsal hair in an area of ~8 cm^2^, 100 *μ*l 1% DNCB dissolved in olive oil was applied onto the dorsal skin once a day to enable sensitization in the first week. The following week, 100 *μ*l 0.2% DNCB was applied to their dorsal skin twice every three days. DNCB was dissolved in a 4:1 mixture of acetone and olive oil. C5aRA JPE-1375 (Jerini AG, Berlin, Germany) 1 *μ*g in 100 *μ*l phosphate-buffered saline (PBS; 137 mM NaCl, 2.7 mM KCl, 4.3 mM Na_2_HPO_4_, 1.4 mM KH_2_PO_4_; pH adjusted to 7.4 with HCl, Sigma-Aldrich, Shanghai, China) was injected intradermally. After a 2-week DNCB application period with or without C5aRA intracutaneous injection, blood samples from the posterior vena cava of the mice were used for immunological or hematological analysis. Furthermore, skin tissue from each mouse examined was sampled, a section was treated by sonication and immediately centrifuged at 1,000 × g for 10 min for ELISA, and the remainder was fixed with 10% neutral buffered formalin solution for histopathological examination.

### Reverse transcription quantitative polymerase chain reaction (RT-qPCR)

Following DNCB application for 2 weeks, total RNA was extracted from skin tissue and relative levels of messenger ribonucleic acid (mRNA) expression of C5aR were normalized to 18s. The following primers were used: for C5aR forward, 5′-GACCCCATAGATAACAGCA-3′ and reverse, 5′-CAGAGGCAACACAAAACCCA-3′; 18s forward, 5′-GTAACCCGTTGAACCCCATT-3′ and reverse, 5′-CCATCCAATCGGTAGTAGCG-3′; and GAPDH forward, 5′-TTGCCATCAATGACCCCTTCA-3′ and reverse, 5′-CGCCCCACTTGATTTTGGA-3′. RT-qPCR was performed using a PrimeScript™ RT reagent kit (Takara Bio, Inc., Tokyo, Japan) with a Veriti™ Thermal Cycler (Applied Biosystem, Foster City, CA, USA) at 37°C for 15 min, 85°C for 5 sec and maintained at 4°C; and an SYBR Premix Ex Taq kit (Takara Bio, Inc.) using a 7300 Fast Real-Time PCR system (Applied Biosystems) under cycling conditions of 95°C for 30 sec for one cycle and 40 cycles of 95°C for 5 sec and 60°C for 31 sec.

### Measurement of skin-fold thickness and histological analysis

Skin-fold thickness was measured using a digital thickness gauge (Mitutoyo, Kawasaki, Japan) by pulling up the skin from shoulder to hip. The skin biopsies were fixed with 10% neutral buffered formalin solution (~4% formaldehyde) and then embedded in paraffin. Paraffin sections (3 *μ*m each; Sigma-Aldrich) were stained with hematoxylin and eosin (H&E) solution for detecting skin histological features and various inflammatory cells (mast cells, neutrophils, lymphocytes, monocytes and a few eosinophils and basophils) and toluidine blue solution was used for detecting mast cells. In brief, the skin sections were washed with distilled water, stained with the alum hematoxylin (Sigma-Aldrich), rinsed in running tap water and then treated with 0.3% hydrochloric acid alcohol (Baowanchem, Nantong, China). The sections were then rinsed under running tap water again and then washed in Scott’s tap water substitute (RY Bio-tech Co., Shanghai, China) prior to rinsing again in tap water. Sections were stained with eosin (Sigma-Aldrich) for 2 min and then dehydrated in 95% and absolute alcohols, two changes of 2 min each until excess eosin was cleared, and mounted. The microscopy images were prepared using an automatic microscope, Provis AX (Olympus, Tokyo, Japan), and a digital CCD camera, Penguin 600CL (Pixera, Los Gatos, CA, USA). For toluidine blue solution for mast cells, the skin sections were hydrated with distilled water, stained in toluidine blue for 2–3 min, washed in distilled water 3 times, dehydrated quickly through 95% and 2 changes of 100% alcohol, and then cleared in xylene or xylene substitute with 2 changes of 3 min each. For morphometric analysis, digital images were captured of three different areas at least at a microscopic high-power field and the numbers of infiltrating leukocytes (objective lens, ×100) and mast cells (objective lens, ×400) were counted as described previously (14).

### Immunological observation

Blood from the posterior vena cava of mice and skin tissue from each mouse examined were sampled and used for immunological analysis. Production of IL-4 and IFN-γ in skin tissue and IL-4, IFN-γ, histamine and IgE levels in serum were determined using ELISA kits (Mouse IL-4, Mouse IFN-γ, Porcine Histamine HIS and Mouse IgE ELISA kits; Sigma-Aldrich) according to the manufacturer’s instructions. The absorbance at 450 nm was measured using an ELISA reader (MTP-800; Corona Electric Co., Ltd, Tokyo, Japan).

### Statistical analysis

All statistical analyses were performed using SPSS 19.0 software (International Business Machines, Armonk, NY, USA). Values are expressed as the mean ± standard error of the mean or the mean ± standard deviation. Each experiment was repeated at least three times. Student’s t-test was used and P<0.05 was considered to indicate a statistically significant difference.

## Results

### C5aR expression significantly increases in skin tissue of mice with AD

**Figure 1:**
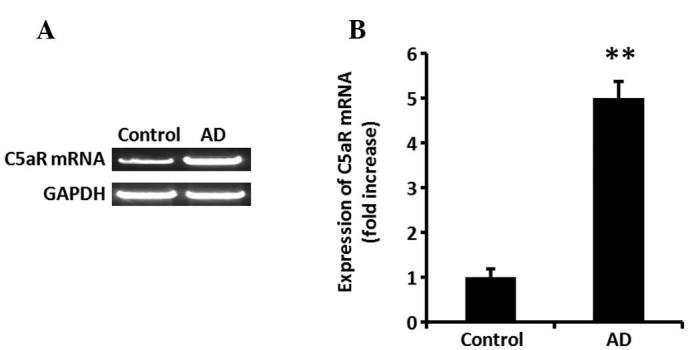
C5aR expression significantly increases in AD mouse skin tissue. To investigate the expression of C5aR, BALB/c mice were treated with DNCB on the dorsal skin. Following DNCB application for 2 weeks, mRNA expression of C5aR in mouse tissue was assessed by reverse transcription quantitative polymerase chain reaction. (A) C5aR expression was significantly increased in AD mouse skin tissue. (B) Quantification of C5aR expression. Values are expressed as the mean ± standard deviation (n=5). ^**^P<0.01, AD vs. control. AD, atopic dermatitis; DNCB, 2,4-dinitrochlorobenzene; C5aRA, C5a receptor antagonist.

To investigate the expression of C5aR, BALB/c mice were treated with DNCB. Following DNCB application onto dorsal skin for 2 weeks, the mRNA expression of C5aR in the tissue was assessed using RT-qPCR. C5aR expression was significantly increased in AD mouse skin tissue (Fig. 1A and B; P<0.01).

### C5aRA reduces the increased skin-fold thickness in mice with AD

**Figure 2:**
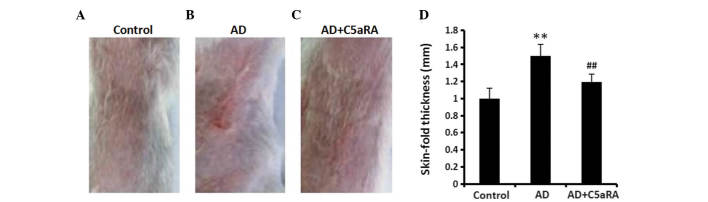
C5aRA reduces the increased skin-fold thickness in mice with AD. DNCB was applied onto the dorsal skin of BALB/c mice for 2 weeks with or without C5aRA (1 *μ*g) intracutaneous injection. Skin-fold thickness was measured using a digital thickness gauge. The skin-fold thickness was significantly increased in mice with AD, which was significantly attenuated by intracutaneous C5aRA injection. Images of the dorsal skin of mice in (A) the control, (B) AD and (C) AD+C5aRA groups (Magnification, ×10). (D) Quantification of A, B and C. Values are expressed as the mean ± standard error of the mean (n=5). ^**^P<0.01, AD vs. control; ^##^P<0.01, AD+C5aRA vs. AD. AD, atopic dermatitis; DNCB, 2,4-dinitrochlorobenzene; C5aRA, C5a receptor antagonist.

DNCB was applied onto BALB/c mouse dorsal skin for 2 weeks with or without C5aRA intracutaneous injection. Skin-fold thickness was measured using a digital thickness gauge. The skin-fold thickness was significantly increased in mice with AD (Fig. 2; P<0.01) which was significantly attenuated in the mice that had received intracutaneous C5aRA injections (P<0.01). C5aRA intracutaneous injection alone did not affect the skin-fold thickness in normal mice (data not shown).

### C5aRA attenuates inflammatory cell infiltration in skin tissue of mice with AD

**Figure 3:**
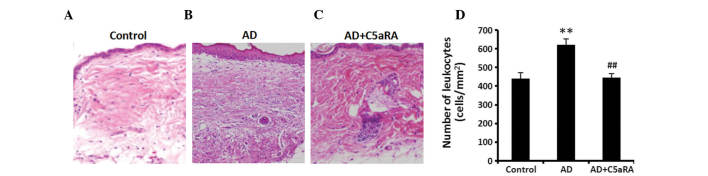
C5aRA attenuates inflammatory cell infiltration in the skin tissue of mice with AD. DNCB was applied onto the dorsal skin of BALB/c mice for 2 weeks with or without C5aRA (1 *μ*g) intracutaneous injection. Following DNCB application, skin tissue from each mouse examined was sampled and fixed with 10% neutral buffered formalin solution. Skin was stained with hematoxylin and eosin solution to detect histological features and various inflammatory cells. The number of infiltrating cells (mast cells, neutrophils, lymphocytes, monocyte and a few eosinophils and basophils) in skin tissue was counted at a microscopic high-power field (objective lens, ×100). The number of infiltrating cells in skin tissue was significantly increased in AD mice, which was significantly attenuated following intracutaneous C5aRA injection. Images of inflammatory cell infiltration in skin tissue of mice in (A) control, (B) AD and (C) AD+C5aRA groups (magnification, ×20). (D) Quantification of A, B and C. Values are expressed as the mean ± standard deviation (n=5). ^**^P<0.01, AD vs. control; ^##^P<0.01, AD+C5aRA vs. AD. AD, atopic dermatitis; DNCB, 2,4-dinitrochlorobenzene; C5aRA, C5a receptor antagonist.

**Figure 4:**
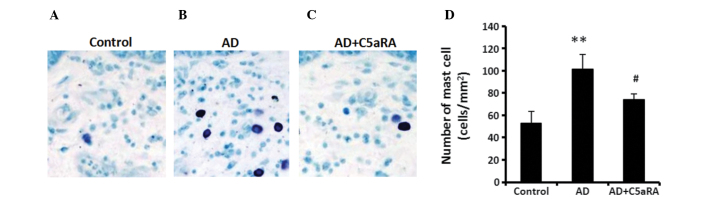
C5aRA attenuates mast cell infiltration in AD mouse skin tissue. Following application of DNCB onto the dorsal skin of BALB/c mice for 2 weeks with or without C5aRA (1 *μ*g) intracutaneous injection, skin tissue from each mouse examined was sampled and fixed with 10% neutral buffered formalin solution. Skin was stained with toluidine blue solution for detecting mast cells, which were counted in a microscopic high-power field (objective lens, ×400). The number of mast cells in skin tissue was significantly increased in AD mice, which was significantly attenuated in the mice receiving C5aRA intracutaneous injection. Images of mast cells in the skin tissue of mice in (A) control, (B) AD and (C) AD+C5aRA groups (magnification, ×200). (D) Quantification of A, B and C. Values are expressed as the mean ± standard deviation (n=5). ^**^P<0.01, AD vs. control; ^#^P<0.05, AD+C5aRA vs. AD. AD, atopic dermatitis; DNCB, 2,4-dinitrochlorobenzene; C5aRA, C5a receptor antagonist.

DNCB was applied onto BALB/c mouse dorsal skin for 2 weeks with or without C5aRA intracutaneous injection. Following DNCB application, skin tissue from each mouse examined was sampled and fixed with 10% neutral buffered formalin solution. Skin was stained with H&E solution for detecting histological features and various inflammatory cells and toluidine blue solution for detecting mast cells. The numbers of total infiltrating leukocytes (mast cells, neutrophils, lymphocytes, monocytes and a few eosinophils and basophils) and mast cells in skin tissue were counted in a microscopic high-power field. The numbers of infiltrating leukocytes and mast cells in skin tissue were significantly increased in mice with AD (Figs. 3 and 4; P<0.01), while the increased skin-fold thickness as well as numbers of infiltrating leukocytes (Fig. 3; P<0.01) and mast cells (Fig. 4; P<0.05) were significantly decreased in the mice that had received intracutaneous C5aRA injections.

### C5aRA attenuates DNCB-mediated increases in IL-4 and IFN-γ levels in skin tissue

**Figure 5:**
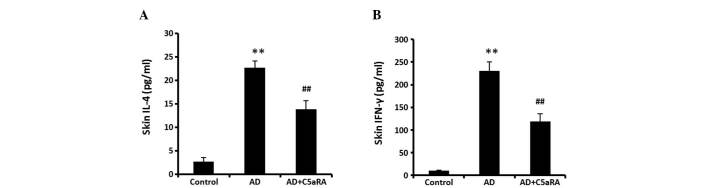
C5aRA reduces the increased IL-4 and IFN-γ levels in mouse skin tissue. BALB/c mice were treated with DNCB on dorsal skin for 2 weeks with or without C5aRA (1 *μ*g) intracutaneous injection. Following DNCB application, the levels of IL-4 and IFN-γ were determined using ELISA kits. (A) Levels of IL-4 in skin tissue were significantly increased. This increase in IL-4 was significantly attenuated in the mice receiving C5aRA intracutaneous injection. (B) Levels of IFN-γ in skin tissue was significantly increased. The increases in IFN-γ levels were significantly attenuated in the mice receiving C5aRA intracutaneous injection. Values are expressed as the mean ± standard deviation (n=5). ^**^P<0.01, AD vs. control; ^##^P<0.01, AD+C5aRA vs. AD. AD, atopic dermatitis; DNCB, 2,4-dinitrochlorobenzene; IL, interleukin; IFN, interferon; C5aRA, C5a receptor antagonist.

BALB/c mice were administered DNCB onto the dorsal skin for 2 weeks with or without C5aRA intracutaneous injection. Following DNCB application, the levels of IL-4 and IFN-γ were determined using ELISA kits. The levels of IL-4 and IFN-γ in skin tissue were significantly increased (Fig. 5, P<0.01). However, these increases in levels of IL-4 and IFN-γ were significantly attenuated in the mice receiving C5aRA intracutaneous injection (P<0.01).

### C5aRA reduces the levels of IL-4, IFN-γ, histamine and IgE in serum

**Figure 6:**
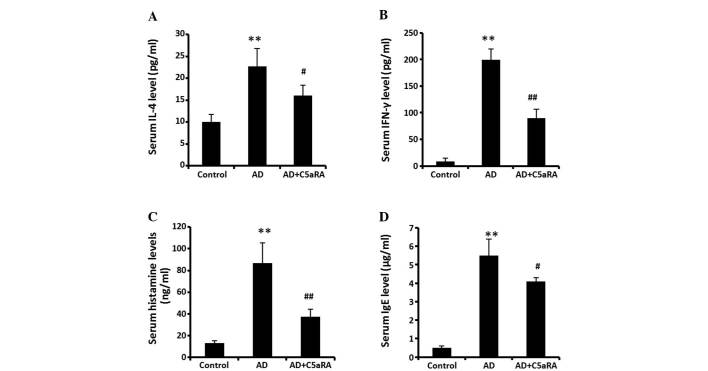
C5aRA reduces the levels of IL-4, IFN-γ, histamine and IgE in mouse serum. Following DNCB application onto the dorsal skin of BALB/c mice for 2 weeks with or without C5aRA (1 *μ*g) intracutaneous injection, blood was sampled from the posterior vena cava of mice. Levels of IL-4, IFN-γ, histamine and IgE in serum were determined using ELISA kits. Levels of (A) IL-4, (B) IFN-γ, (C) histamine and (D) IgE were significantly increased. The increases in levels of IL-4, IFN-γ, histamine and IgE in serum were significantly attenuated in the mice receiving C5aRA intracutaneous injection. Values are expressed as the mean ± standard deviation (n=5). ^**^P<0.01, AD vs. control; ^##^P<0.01, ^#^P<0.05, AD+C5aRA vs. AD. AD, atopic dermatitis; DNCB, 2,4-dinitrochlorobenzene; Th, T helper; IL, interleukin; IFN, interferon; IgE, immunoglobulin E; C5aRA, C5a receptor antagonist.

DNCB was applied onto the dorsal skin of BALB/c mice for 2 weeks with or without C5aRA intracutaneous injection. Following DNCB application, blood was sampled from the posterior vena cava of the mice. The levels of IL-4, IFN-γ, histamine and IgE in serum were significantly increased (Fig. 6; P<0.01). The increased levels of IL-4 (P<0.05), IFN-γ (P<0.01), histamine (P<0.01) and IgE (P<0.05) in serum were significantly decreased in the mice receiving intracutaneous injection of C5aRA.

## Discussion

In the present study, it was demonstrated for the first time, to the best of our knowledge, that C5aRA inhibits AD in mice by suppressing the C5aR-mediated cascade action of mast cells. AD is an inflammatory skin disease that is associated with allergies and genetics (1). Atopic dermatitis occurs mainly from infiltration of various inflammatory cells (mast cells, neutrophils, lymphocytes, monocytes and a few eosinophils and basophils), particularly mast cells (2) and dysregulation in Th cell reactivities (3,15). Previously, studies on the complement anaphylatoxin receptors have been undertaken in multiple fields, including cardiovascular disease and inflammation (9–11) and it has been reported that anaphylatoxin receptor expression is involved in skin inflammation (8). C5a specifically binds to its receptor C5aR on immune cells, including mast cells and leads to proinflammatory activation (12). It has been demonstrated that C5a can induce chemoattraction and secretion in mast cells in inflammation through a C5aR-mediated pathway (13). As C5a is a risk factor for skin inflammation and the expression of C5aR was significantly increased in AD mouse skin tissue, the present study further investigated whether C5aRA inhibited AD in mice by suppressing the C5aR-mediated cascade action of mast cells.

AD is an inflammatory skin disease and is distinguished by an increase in skin-fold thickness and infiltration of numerous inflammatory cells into the site of inflammation (1,2). In the present study, following DNCB application onto the BALB/c mouse dorsal skin for 2 weeks, the skin-fold thickness was significantly increased in AD mice and in addition, the increase in skin-fold thickness was significantly reduced in the mice that had received C5aRA intracutaneous injection. Similarly, the quantity of total infiltrating leukocytes (mast cells, neutrophils, lymphocytes, monocytes and a few eosinophils and basophils) and mast cells in the skin tissue were significantly increased in mice with AD, which was significantly attenuated in the mice that had received C5aRA intracutaneous injection.

IL-4, which is produced by Th2 cells and mast cells (4,16) and IFN-γ, which is produced by Th1 or mixed Th1/Th2 cells, are known to trigger isotype-switching to immunoglobulins in AD (6). Excessive IgE production is a hallmark of atopic dermatitis in humans and has been observed in mice with atopic dermatitis-like skin lesions (16,18). The release of histamine from activated mast cells is an indicator of the occurrence of allergic diseases, including atopic dermatitis, which causes itching, increased vascular permeability and the wheal-and-flare response of immediate hypersensitivity (19). As histamine is released immediately following exposure to allergens, the increase in serum histamine levels is considered a parameter for diagnosing the onset of allergic diseases (6,20). In the present study, it was identified that in skin tissue, the levels of IL-4 and IFN-γ were significantly increased in mice with AD. The increases in levels of IL-4 and IFN-γ were significantly attenuated in the mice receiving C5aRA intracutaneous injection (Fig. 5). In the serum, the levels of IL-4, IFN-γ, histamine and IgE were also significantly increased, which was significantly attenuated in the mice receiving C5aRA intracutaneous injection. Results of serum analysis confirmed the findings in skin tissue.

**Figure 7:**
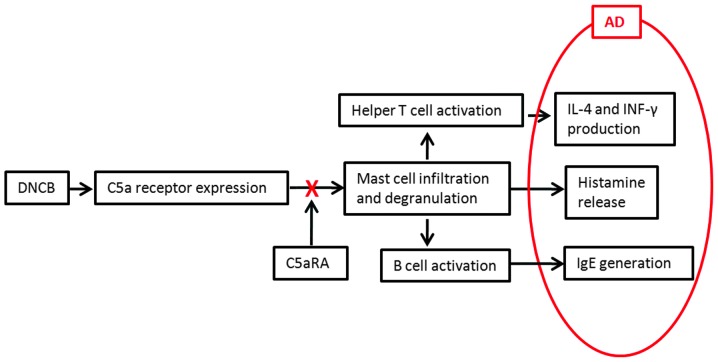
Inhibitory mechanism of C5aRA on AD. Following DNCB application onto mouse dorsal skin for 2 weeks, C5aR expression in skin tissue was significantly increased and the infiltration of mast cells was significantly enhanced as well. The activated mast cells released polarizing cytokines to increase IL-4 production from Th2 and IFN-γ production from mixed Th1/Th2. In addition, the increased C5aR expression on mast cells enhanced IgE in B cells. At the same time, mast cells released histamine to generate pruritus symptoms. Intracutaneous injection of C5aRA directly prevented the binding of C5a to C5aR on mast cells, then decreased the skin-fold thickness, number of infiltrating leukocytes and mast cells as well as levels of IL-4, IFN-γ, histamine and IgE, and thereby inhibited the symptoms of AD. AD, atopic dermatitis; DNCB, 2,4-dinitrochlorobenzene; Th, T helper; IL, interleukin; IFN, interferon; IgE, immunoglobulin E; C5aRA, C5a receptor antagonist.

The increased levels of IL-4 and IFN-γ, IgE production and histamine release during the AD period are all closely associated with the infiltration of mast cells to the site of inflammation (2). Mast cells are involved in AD by releasing Th2 and Th1/Th2, polarizing cytokines to increase IL-4 and IFN-γ expression levels, while generating pruritus symptoms through release of histamine and tryptase (7). In addition, mast cells may increase histamine release and enhance IgE-dependent skin inflammation in mice (8). In general, there is a balance between mast cells and other pathogenesis factors of AD. There is also a balance between two types of Th cells and any disruption of the balance may cause a variety of disorders, including AD (21,22). Corresponding with the mechanism shown in Fig. 7, following DNCB application onto mouse dorsal skin for 2 weeks, C5aR expression in skin tissue was significantly increased and the infiltration of mast cells was significantly enhanced as well. C5aR is expressed on multiple types of immune cells, including mast cells in skin tissue. C5a specifically binds to the increased C5aR (23). Subsequently, the binding reaction feeds back to mast cells and induces the chemoattraction of mast cells (24). As shown in Fig. 7, the activated mast cells released polarizing cytokines to increase IL-4 production from Th2 and IFN-γ production from mixed Th1/Th2. In addition, the increased C5aR expression on mast cells enhanced IgE in B cells. At the same time, mast cells released histamine to generate pruritus symptoms. These pathological processes constitute the pathogenesis of AD. Intracutaneous injection of C5aRA directly prevented the binding of C5a to C5aR on mast cells, then decreased the skin-fold thickness, numbers of infiltrating leukocytes and mast cells levels of IL-4, IFN-γ, histamine and IgE, thereby inhibiting the symptoms of AD. The complement receptor cascade is a complex process, although it was demonstrated that C5aRA inhibited AD in mice by suppressing C5aR-mediated cascade action of mast cells. The complex inhibitory process and the intracellular pathway require further investigation.

The present study was the first, to the best of our knowledge, to demonstrate a crucial role of C5a-C5aR-C5aRA action in the pathogenesis of AD, suggesting that C5aRA inhibited AD in mice by suppressing the C5aR-mediated cascade action of mast cells and providing a rationale for the potential use of C5aRA in clinical practice for AD. These observations indicated that C5a-C5aR-C5aRA may represent a novel therapeutic target for AD.
